# 固相萃取-高效液相色谱-三重四极杆质谱法分析饮用水处理厂水样中7种香豆素

**DOI:** 10.3724/SP.J.1123.2024.06014

**Published:** 2025-01-08

**Authors:** Wenmei JIAO, Jingming YANG, Ce XU, Fukang GAO, Luyao SHEN, Yubo YUAN, Zhifen GUO, Guang HUANG

**Affiliations:** 1.江苏第二师范学院生命科学与化学化工学院, 江苏 南京 211200; 1. School of Life Sciences and Chemical Engineering, Jiangsu Second Normal University, Nanjing 211200, China; 2.南京医科大学口腔医学院, 江苏 南京 210029; 2. School of Stomatology, Nanjing Medical University, Nanjing 210029, China; 3.南京医科大学公共卫生学院, 江苏 南京 211166; 3. School of Public Health, Nanjing Medical University, Nanjing 211166, China; 4.南京医科大学公共卫生学院现代毒理学教育部重点实验室, 江苏 南京 211166; 4. Key Laboratory of Modern Toxicology, Ministry of Education, School of Public Health, Nanjing Medical University, Nanjing 211166, China

**Keywords:** 固相萃取, 高效液相色谱-串联质谱法, 饮用水, 香豆素, 消毒副产物, solid-phase extraction (SPE), high performance liquid chromatography-tandem mass spectrometry (HPLC-MS/MS), drinking water, coumarins, disinfection byproducts

## Abstract

本研究建立了一种固相萃取结合高效液相色谱-三重四极杆质谱法分析饮用水处理厂水样中7种香豆素类化合物的方法。样品前处理过程采用HLB小柱进行固相萃取,然后采用超纯水(含0.25%甲酸)淋洗,最后经甲醇(含0.25%甲酸)洗脱。萃取液用Phenomenex Luna C18柱(100 mm×2.0 mm, 3 μm)分离后,以甲醇-0.1%甲酸水溶液为流动相进行梯度洗脱,在电喷雾多反应监测模式下检测。以饮用水原水和出厂水为基质考察方法的基质效应、精密度和准确度。7种香豆素类化合物在各自的线性范围内呈现良好的线性关系,相关系数(*r*)均大于0.99,方法检出限(MDL)为0.67~1.12 ng/L。不同加标水平(20、50、100 ng/L)下7种香豆素类化合物回收率为61.4%~91.5%,相对标准偏差(RSD, *n*=6)均≤11.2%。利用该方法分析了饮用水处理厂不同处理阶段的水样,其中7-羟基香豆素、6,7-二羟基香豆素、香豆素的检出率为100%,含量为0.21~27.9 ng/L。6-羟基-4-甲基香豆素在原水、混凝后水样、炭滤后水样中均未检出,只在砂滤后水样和出厂水中被检出,而且出厂水中的含量(4.69 ng/L)高于砂滤后水样(1.79 ng/L)。8-氯-7-羟基香豆素只在出厂水中被检出(0.28 ng/L)。本方法分析时间短,精密度和准确度良好,在监测实际水样中香豆素的含量及评估饮用水处理过程对于水样中香豆素类化合物去除方面可发挥积极作用。

## Abstract (en)

Chlorinated coumarins, which are as cytotoxic as highly toxic halobenzoquinones toward CHO-K1 cells, have recently been identified as disinfection byproducts in drinking water disinfection processes. Therefore, detecting coumarins in water samples collected at various stages from drinking water treatment plants helps assess the formation of chlorinated coumarins in drinking water. Hence, a simple, rapid, accurate, and sensitive method for quantifying coumarins in water samples is required.

In this study, a method was developed based on solid-phase extraction coupled with high performance liquid chromatography-triple-quadrupole mass spectrometry for analyzing seven coumarins in water samples from drinking water treatment plants, including 6,7-dihydroxycoumarin, 7-hydroxycoumarin, 6-hydroxy-4-methylcoumarin, 8-chloro-7-hydroxycoumarin, coumarin, 7-chloro-6-hydroxy-4-methylcoumarin, and 3,8-dichloro-7-hydroxycoumarin. Sample pretreatment involved solid-phase extraction using HLB columns, followed by elution with water and methanol, each containing 0.25% formic acid. The extracted solution was separated via gradient elution using a Phenomenex Luna C18 column (100 mm×2.0 mm, 3 mm) with 0.1% formic acid aqueous solution and methanol as the mobile phases, with analytes detected by triple-quadrupole mass spectrometry equipped with electrospray ionization source in multiple reaction monitoring mode. The matrix effect, precision, and accuracy of the developed method were investigated using raw and treated water as matrices. Matrix effects of 0.84-1.12 were recorded for the detection of 6,7-dihydroxycoumarin, 7-hydroxycoumarin, 6-hydroxy-4-methylcoumarin, and coumarin in raw water, while values of 0.67-0.70 were recorded for 8-chloro-7-hydroxycoumarin, 7-chloro-6-hydroxy-4-methylcoumarin, and 3,8-dichloro-7-hydroxycoumarin in finished water. The three chlorinated coumarins exhibited matrix effects above 0.80 after the finished water matrix had been diluted four times. These results suggest that only simple solid-phase extraction or sample dilution is required to accurately determine the seven coumarins in drinking water from treatment plants. In addition, these coumarins exhibited good linear relationships at their respective mass concentrations. The precision and accuracy of the method were evaluated using raw and treated water as matrices. The seven coumarins exhibited good linearities by triple-quadrupole mass spectrometry in a certain range, with correlation coefficients (*r*) greater than 0.99 and method detection limits (MDLs) of 0.67-1.12 ng/L. The seven coumarins exhibited recoveries of 61.4%-91.5% at three spiked levels (20, 50, 100 ng/L) with relative standard deviations (RSDs, *n*=6)≤11.2%. The developed method can be used to analyze water samples from various treatment stages of a drinking-water treatment plant. 7-Hydroxycoumarin, 6,7-dihydroxycoumarin, and coumarin were detected at levels of 0.21-27.9 ng/L in 100% of the samples, while 6-hydroxy-4-methylcoumarin was not detected in raw water, post-coagulated water, and post-carbon-filtered water, but was found in sand-filtered water and finished water, with higher levels recorded for the latter (4.69 ng/L) than the former (1.79 ng/L). 8-Chloro-7-hydroxycoumarin was only detected in treated water (0.07 ng/L). This method is highly precise and accurate, provides results in short analysis times, and can be used to effectively monitor coumarins in real water samples and assess their removal during drinking-water treatment.

自然植被或农作物产生的次级代谢产物以渗滤液、根部渗出物的形式通过雨水管道、表面径流以及植物分解的方式释放到环境中^[1]^,最后随降雨转移到地表水中^[2]^;水体中还存在水生植物、细菌、真菌的代谢产物以及腐殖质的分解产物,这些因素的共同作用导致饮用水原水中存在许多天然产物类的溶解性有机物。相关研究发现自然植被或农作物产生的次级代谢产物中含有对人体健康有危害的香豆素类化合物^[3]^。此外,水体中的水生生物中也分离到香豆素成分,如睡莲属的不同物种中均可分离出香豆素等生物活性成分^[4]^。香豆素类化合物具有*α*,*β*-不饱和内酯结构,可与蛋白质和DNA的亲核基团发生反应导致细胞死亡。甲基香豆素类化合物消耗大鼠肝细胞中的谷胱甘肽导致大鼠肝细胞毒性的产生^[5]^。Liu等^[6]^对饮用水原水中的天然产物成分进行非靶向筛查发现香豆素类化合物在原水中含量非常丰富,腐殖质在预氯化处理中容易转化成香豆素类化合物,后续的消毒过程中容易形成氯代香豆素,如3-氯-7-甲氧基-4-甲基香豆素。在评价饮用水中消毒副产物对中国仓鼠卵巢细胞的细胞毒性时发现,3-氯-7-甲氧基-4-甲基香豆素的毒性贡献(0.21%)与饮用水中高毒性消毒副产物2,6-二氯-1,4-对苯二醌(0.25%)和2,6-二溴-1,4-对苯二醌(0.51%)的毒性贡献相当。由于香豆素类化合物通常具有强烈的芳香气味,它们常常用于护肤品中,如香皂、沐浴露、洗涤剂、乳液和护发素,并随这些日用品的使用进入到水体,导致水体中香豆素类化合物的含量增加。此外,Blanco等^[7]^报道香豆素可作为内分泌干扰剂,诱导鱼类的雌激素反应,上述结果说明水体中的香豆素类化合物不仅影响人体健康,还可能危及生态环境安全。因此,监测水体中的香豆素含量有助于了解人群通过接触水体方式暴露于香豆素类化合物的情况。

目前香豆素类化合物在各类样品中的含量测定方法通常包括高效液相色谱法、气相色谱法、高效液相色谱-串联质谱法、气相色谱-串联质谱法^[8]^。其中高效液相色谱法在分析植物提取物中的香豆素成分时应用较多,如分析草本植物草木樨或者北沙参中香豆素的成分及含量^[9]^,这主要是因为液相色谱法简单方便,而且检测目标均为一些有标准品的活性成分,但是液相色谱仪的检出限不如质谱仪。植物提取物中香豆素活性成分的检测可以用液相色谱法分析是因为这些植物的质量足够多,提取液中香豆素含量较高,能够通过液相色谱法检出。气相色谱法和气相色谱-串联质谱法分析样品时样品前处理需要用到大量有机溶剂,前处理过程相对繁琐,尤其在检测沸点较高的香豆素时,色谱柱流失产生的噪声可干扰检测,且对沸点相近的香豆素无法实现较好分离,因而定量准确性不佳^[10]^。样品前处理结合高效液相色谱-串联质谱法适合分析复杂基质中的痕量样品,能有效弥补上述方法存在的局限性。实际上水体中香豆素类物质的含量相对较低(ng/L水平),经富集后才能被液相色谱-串联质谱准确和灵敏地检测^[11]^。目前水样中消毒副产物富集的经典方式有液液萃取和固相萃取^[12]^,其中液液萃取需要用大量的有机溶剂,容易发生乳化现象,萃取时间长,不利于环境保护和操作人员的安全,而固相萃取适合处理大体积的样品,更为简便、快速,易于实现自动化,能有效地减弱基质的干扰作用。尽管在固相萃取和液液萃取的基础上开发出液相微萃取和固相微萃取技术,但是液相微萃取过程中液滴不稳定或者液滴的损失容易造成回收率低和重现性差,而固相微萃取技术中很多研究只是发展了新的固相微萃取吸附剂,但这些新吸附剂一般都是针对特定的分析物开发的,广谱性较差,离真正的应用有一定距离^[13]^。固相萃取技术较为成熟,而且有多种商品化的固相萃取小柱可供选择,能有效地保证实验结果的重复性。

针对上述问题,本研究利用固相萃取结合高效液相色谱-串联质谱分析建立了饮用水水样中7种香豆素类化合物的分析方法,并用于实际水样的分析。该方法的建立对于准确监测饮用水处理厂水样中香豆素类化合物和评估饮用水处理厂净水单元对饮用水水质影响具有重要意义。

## 1 实验部分

### 1.1 仪器与试剂

UltiMate 3000液相色谱-Q Exactive混合四极杆-轨道阱质谱仪(美国赛默飞世尔科技公司);ExionLC AD液相色谱-Sciex 6500三重四极杆质谱仪(美国Sciex科技公司);干式氮吹仪(广州恒美仪器有限公司)。

质谱级甲醇、乙腈、甲酸购自天津市科密欧化学试剂有限公司,Supelco固相萃取装置购自美国赛默飞世尔科技公司,HLB固相萃取柱(500 mg/6 mL)、C18固相萃取柱(500 mg/6 mL)购自苏州纳微科技股份有限公司。娃哈哈纯净水购自当地超市。

标准品:7-羟基香豆素(纯度98%)、6-羟基-4-甲基香豆素(纯度99%)、6,7-二羟基香豆素(纯度98%)、香豆素(纯度99%)购自实验谷一站式采购平台;8-氯-7-羟基香豆素(纯度92%)、3,8-二氯-7-羟基香豆素(纯度94%)、7-氯-6-羟基-4-甲基香豆素(纯度93%)由本实验室合成,具体合成方法见文献[6]。

### 1.2 实验条件

#### 1.2.1 标准溶液配制

用甲醇溶解并配制50 mg/L的7种香豆素标准储备液,于-20 ℃冰箱中保存。

用20%甲醇水溶液稀释上述标准储备液,分别配制0.01、0.1、0.2、0.5、1、2.5、5、7.5、10、25、50、100 μg/L 7种香豆素混合标准溶液。

#### 1.2.2 样品采集与前处理

南京市某饮用水处理厂的水处理工艺为原水依次经混凝、炭滤、砂滤处理后,最后由液氯消毒。采集该水厂各阶段水样4 L,向水样中加入10 mL甲酸以淬灭其中残留的消毒剂。采集的水样先经过90 mm滤纸过滤2次后,再用0.45 μm水系膜过滤2次。采用HLB小柱对过滤后水样进行固相萃取,用12 mL甲醇(含0.25%甲酸)和12 mL纯净水(含0.25%甲酸)活化固相萃取小柱。1 L水样以6 mL/min的速度上样后,先用12 mL纯净水(含0.25%甲酸)淋洗,淋洗后用氮气吹干固相萃取小柱,再用10 mL甲醇(含0.25%甲酸)洗脱。洗脱液在25 ℃下氮吹至0.1 mL,然后向其中加入0.9 mL纯净水(含0.1%甲酸),涡旋均匀后用0.22 μm水系膜过滤,再用HPLC-MS/MS分析。

#### 1.2.3 分析方法

色谱柱:Phenomenex Luna C18柱(100 mm×2.0 mm, 3 μm),柱温:40 ℃;流动相A为0.1%甲酸水溶液,B为甲醇,流速为0.3 mL/min;进样量:10 μL。梯度洗脱程序:0~5 min, 80%A~10%A; 5~7.5 min, 10%A; 7.5~7.51 min, 10%A~80%A; 7.51~10 min, 80%A。

采用Q Exactive混合四极杆-轨道阱质谱仪比较样品中目标物和标准品的精确质荷比、保留时间、碎片离子,快速评价流动相和固相萃取小柱对样品中目标物分析的影响。电喷雾离子源(ESI),正、负离子模式下分别进行全扫描(*m/z* 60~1000)和数据依赖采集(dd-MS^2^),其中dd-MS^2^的碰撞能量分别设置为35、45和55 V。毛细管温度250 ℃,辅助气体加热温度50 ℃,喷雾电压3.5 kV,鞘气5 arb,辅助气体3 arb和反吹气0 arb。Xcalibur软件(2.2版)进行数据分析。

Sciex 6500三重四级杆质谱仪用于检测实际水样中7种香豆素。电喷雾离子源,离子喷雾电压5500 V/-4500 V;离子源温度500 ℃;雾化气压力为310 kPa (45 psi);辅助气压力为276 kPa (40 psi);气帘气压力为207 kPa (30 psi);每对离子的积累时间为100 ms。7种香豆素在多反应监测(MRM)模式下的质谱参数见表1。

**表1:** 7种香豆素类化合物的质谱参数

No.	Compound	Retention time/min	Precursor ion (*m/z*)	Product ion (*m/z*)	DP/ V	CE/ V	CXP/ V
1	6,7-dihydroxycoumarin (6,7-二羟基香豆素)	1.65	179.2	123.1^*^	147.4	30.0	12.6
				133.1	159.6	26.6	16.0
				77.1	141.1	43.9	8.9
2	7-hydroxycoumarin (7-羟基香豆素)	2.61	163.1	107.1^*^	134.6	28.7	13.5
				77.1	136.7	43.0	7.2
				91.1	235.2	47.0	11.4
3	6-hydroxy-4-methylcoumarin (6-羟基-4-甲基香豆素)	2.77	177.2	77.0^*^	141	44.2	18.6
				103.1	153.8	35.5	12.7
				91.1	157.1	37.7	12.6
				121.1	154.0	27.0	10.4
4	8-chloro-7-hydroxycoumarin (8-氯-7-羟基香豆素)	3.19	194.8	166.9^*^	-58.0	-27.6	-12.6
				138.9	-56.5	-30.6	-12.5
				103.0	-46.3	-35.5	-10.8
5	coumarin (香豆素)	3.50	146.9	102.9	79.8	23.8	11.4
				90.9^*^	74.8	32.5	10.5
				76.9	64.8	33.0	35.5
6	7-chloro-6-hydroxy-4-methylcoumarin (7-氯-6-羟基-4-甲基香豆素)	3.51	208.8	173.2^*^	-60.0	-22.0	-16.0
				144.9	-60.0	-30.0	-27.3
7	3,8-dichloro-7-hydroxycoumarin (3,8-二氯-7-羟基香豆素)	4.03	228.9	200.8^*^	-92.6	-29.5	-17.4
				172.8	-52.4	-33.0	-18.5
				194.0	-52.8	-30.2	-17.1

DP: declustering potential; CE: collision energy; CXP: cell exit potential; * quantitative ion.

## 2 结果与讨论

### 2.1 流动相的选择

香豆素类化合物属于极性化合物,ESI源适合分析极性化合物,因而选择ESI源对其进行分析。反相色谱在液相色谱-串联质谱分析中相对常见,色谱分离时0.1%甲酸水溶液-乙腈和0.1%甲酸水溶液-甲醇是最常见的色谱流动相,因为它们的背景吸收低,而且洗脱能力较强。在分析方法建立时,需要评估不同流动相对7种香豆素类化合物离子化信号的影响。图1为0.1%甲酸水-乙腈和0.1%甲酸水-甲醇为流动相时50 μg/L香豆素类化合物在正、负离子扫描模式下的提取离子流色谱图。由图1可知,尽管正离子模式下检出的香豆素化合物数量(7种)多于负离子模式(6种),但是负离子模式下7-羟基香豆素、8-氯-7-羟基香豆素、7-氯-6-羟基-4-甲基香豆素、3,8-二氯-7-羟基香豆素响应高于其正离子模式下的信号,因此在建立高灵敏的分析方法时要针对正、负离子采集模式分别建立方法。总体而言,7种香豆素类化合物在相同的离子模式下0.1%甲酸水-乙腈为流动相的响应均低于0.1%甲酸水-甲醇的响应,主要的原因可能是流动相中的甲醇含有氧原子,在ESI源中可以促进离子的产生,而乙腈中不含氧原子,不利于离子的产生。因此后续分析时主要采用0.1%甲酸水-甲醇为流动相进行分析。本方法中0.1%甲酸水-甲醇作为流动相的另一个特点是甲醇价格相对乙腈而言较为便宜且毒性小,这在实际应用中有利于降低分析成本。

**图1:**
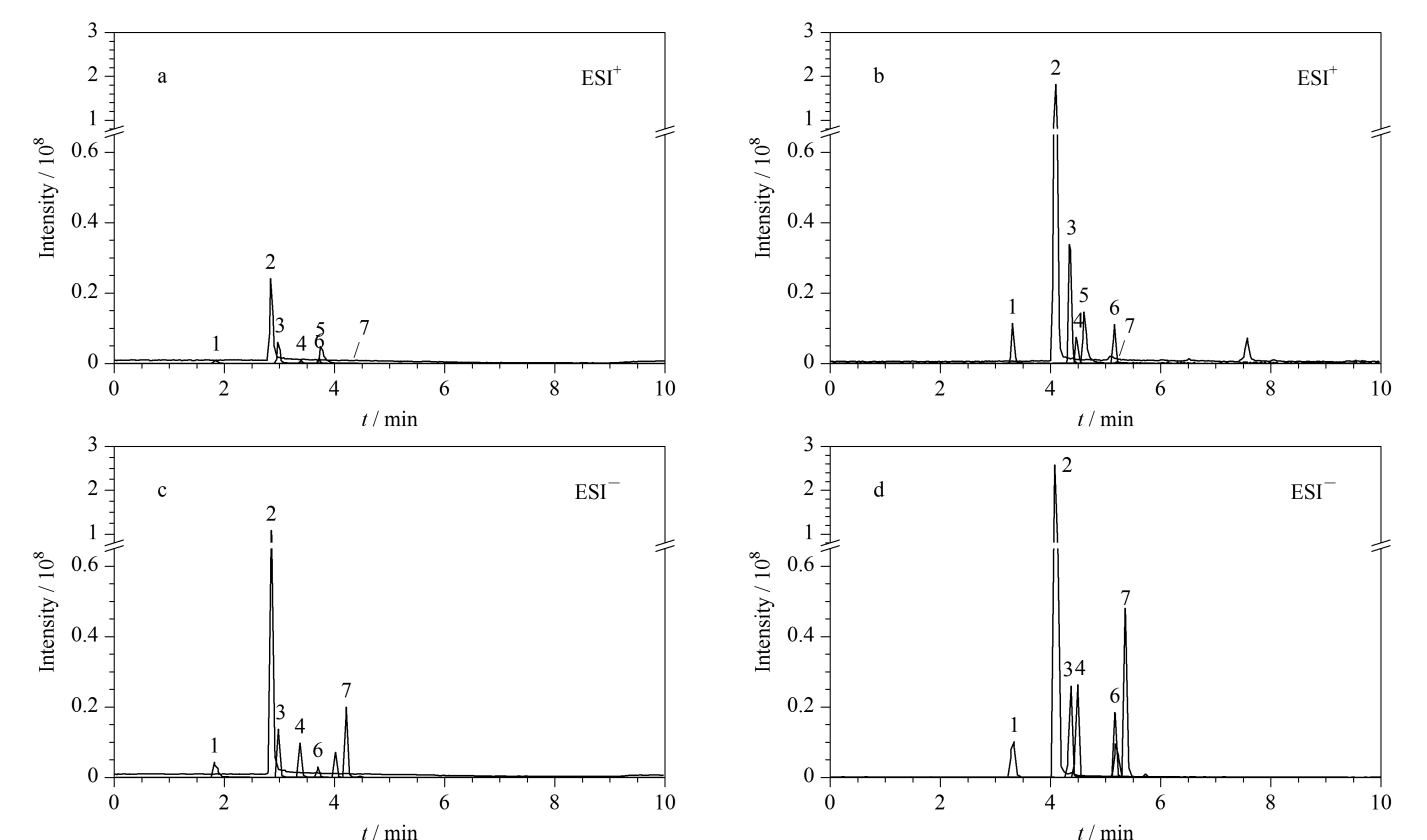
采用不同流动相时7种香豆素(50 μg/L)的提取离子流色谱图

### 2.2 固相萃取小柱的选择

由于实际水样中香豆素类化合物的浓度较低,实际样品分析时往往需要对水样进行固相萃取处理,因此有必要评价固相萃取小柱的萃取效率。HLB固相萃取小柱由亲水亲脂平衡填料组成,填料中含有特定比例的亲水基和疏水基,其中亲水基为*N*-乙烯基吡咯烷酮,可以保留极性化合物,疏水基为二乙烯基苯可保留非极性化合物,因此HLB固相萃取小柱能有效地富集样品中的极性和非极性化合物。C18固相萃取小柱由表面修饰有十八烷基的硅胶微球组成,硅胶微球表面有硅羟基,因而在具有疏水性的同时也有较弱的亲水性。这些香豆素类化合物是含芳环的有机小分子,取代基不同可能导致疏水性有所不同,而HLB小柱和C18小柱可兼顾不同疏水性物质的富集,因此本研究中选择了这两种固相萃取小柱进行评价。向500 mL pH 7的磷酸缓冲盐中加入7种香豆素,评价它们在HLB(500 mg/6 mL)和C18(500 mg/6 mL)小柱上的回收率(见图2)。

**图2:**
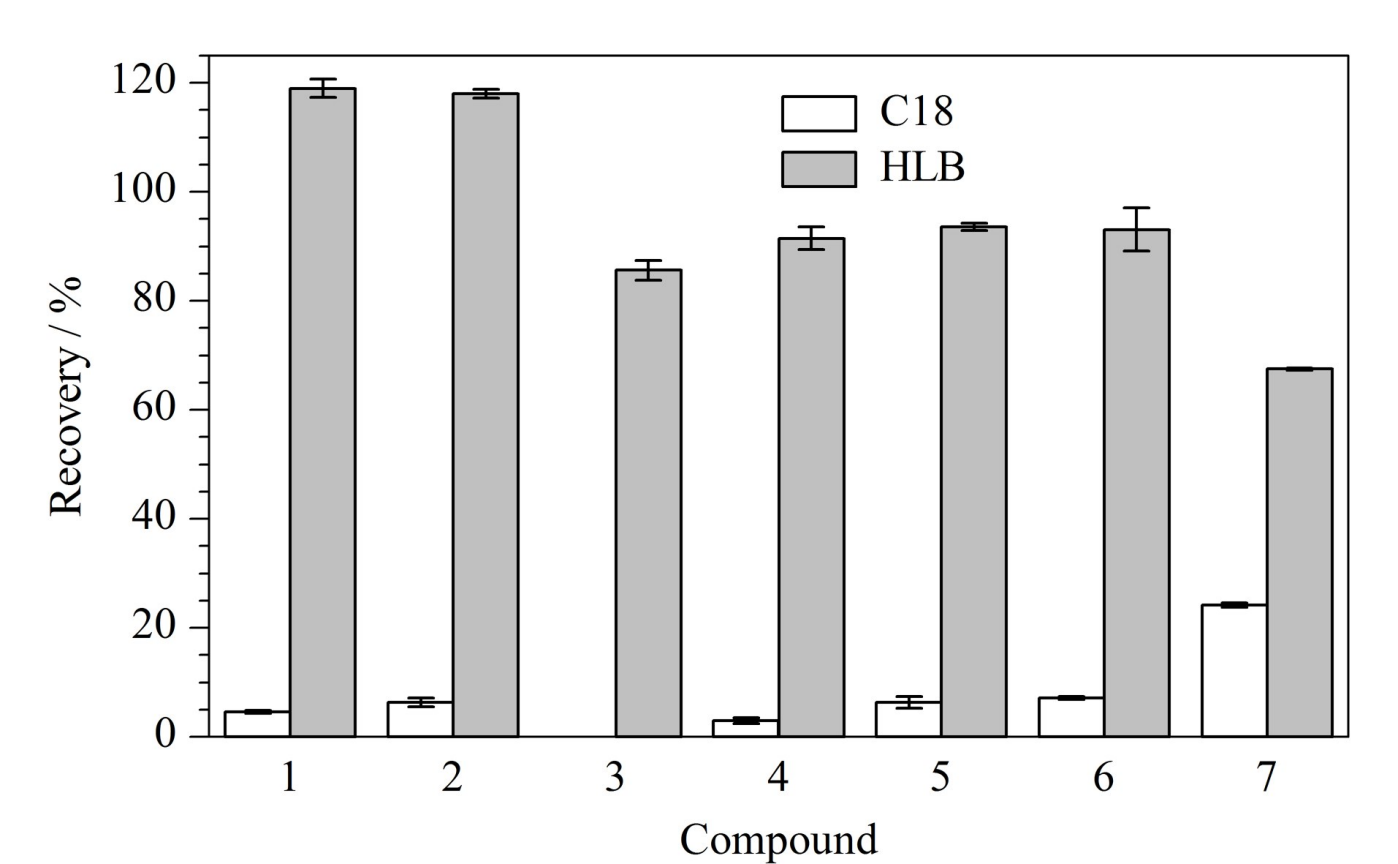
采用不同固相萃取小柱时7种香豆素类化合物的回收率(*n*=3)

对C18固相萃取小柱而言,回收率大小顺序:7-氯-6-羟基-4-甲基香豆素(24.2%)>3,8-二氯-7-羟基香豆素(7.1%)> 8-氯-7-羟基香豆素(6.3%)>6-羟基-4-甲基香豆素(6.2%)>7-羟基香豆素(4.6%)>香豆素(3.0%)>6,7-二羟基香豆素(0),这与Chemspider数据库中给出脂水分配系数的对数值(log *P*)的变化趋势一致。因为log *P*已经被证明可以反馈化合物的疏水性,一般log *P*越大,疏水性越强^[14]^,因而在C18固相萃取小柱中保留越强,回收率也越高。这7种香豆素类化合物在HLB小柱中的回收率与C18柱中不一样,7-羟基香豆素(119%)>6-羟基-4-甲基香豆素(118%)>8-氯-7-羟基香豆素(93.6%)>3,8-二氯-7-羟基香豆素(93.1%)>香豆素(91.5%)>6,7-二羟基香豆素(85.6%)>7-氯-6-羟基-4-甲基香豆素(67.5%),可能是这些香豆素类化合物还具有一定的亲水性,HLB固相萃取小柱中的两亲性基团有利于富集这7种香豆素类化合物。鉴于这7种香豆素类化合物在HLB上的回收率均高于C18小柱,因此后续实际样品分析中采用HLB固相萃取小柱对样品进行固相萃取。

### 2.3 基质效应

实际水样成分比较复杂,往往含有腐殖质等天然有机物,这些天然有机物在天然水体中还可进一步降解,从而加剧实际水样的复杂程度。由于这些天然有机物及其降解产物的化学结构并未得到彻底解析,在HPLC-MS分析饮用水中的消毒副产物时,这些物质可能会在色谱分离中和消毒副产物一起被洗脱,有可能产生基质效应从而干扰消毒副产物的分析。因此,基质效应的评价十分必要。以纯溶剂(20%甲醇水溶液)配制7种香豆素的混合溶液,其中7-羟基香豆素、6-羟基-4-甲基香豆素、6,7-二羟基香豆素和香豆素的质量浓度为15 μg/L, 8-氯-7-羟基香豆素、3,8-二氯-7-羟基香豆素和7-氯-6-羟基-4-甲基香豆素的质量浓度为6 μg/L。以原水固相萃取液为基质,配制15 μg/L 7-羟基香豆素、6-羟基-4-甲基香豆素、6,7-二羟基香豆素和香豆素,以出厂水固相萃取液为基质配制6 μg/L 8-氯-7-羟基香豆素、3,8-二氯-7-羟基香豆素和7-氯-6-羟基-4-甲基香豆素。这主要因为氯代香豆素往往在消毒后的水样中被检出,原水中往往不含氯代香豆素。根据Matuszewski等^[15]^的方法,通过比较化合物在基质溶液和纯溶剂中的信号响应,计算基质效应,具体公式如下。


(1) $$ME=\frac{A_{2}-A_{0}}{A_{1}}$$

式中:*A*_2_指基质加标后香豆素类化合物的峰面积,*A*_0_指基质中香豆素类化合物的峰面积,*A*_1_指纯溶剂中香豆素类化合物的峰面积。基质效应为0.8~1.2时,基质效应对目标物的影响可以忽略,而基质效应为0.5~0.8或者1.2~1.5时,基质效应对目标物具有抑制或者增强作用^[16]^。由表2可知,对于原水中的4种香豆素而言,它们的基质效应为0.84~1.12,因此测量原水中这4种香豆素时,可以不用考虑基质效应。而对于出厂水中的3种氯代香豆素而言,基质效应为0.67~0.70,据Ferrer等^[17]^报道基质效应存在时,对样品进行稀释可有效减少基质效应问题。后续测量出厂水中3种氯代香豆素时,对基质分别稀释2、4、8倍,发现基质稀释4倍和8倍后基质效应为0.81~0.99(表2),表明4倍以上的稀释有效降低了基质效应的影响,可直接检测其中的氯代香豆素。鉴于本研究中建立的固相萃取方法在上样结束后,只包括水(含0.25%甲酸)淋洗和甲醇(含0.25%甲酸)洗脱2个步骤,具有步骤少、富集时损失小的特点,而且通过直接检测或者稀释固相萃取后的样品可满足7种香豆素类化合物的检测,因此该固相萃取方法不繁琐,具有较好的实际应用前景。

**表2:** 实际水样中基质稀释不同倍数后7种香豆素类化合物的基质效应

Compound	Matrix effects							
	Without dilution		2-fold dilution		4-fold dilution		8-fold dilution	
7-Hydroxycoumarin		0.88		NA		NA		NA
6-Hydroxy-4-methylcoumarin		0.92		NA		NA		NA
6,7-Dihydroxycoumarin		1.12		NA		NA		NA
Coumarin		0.84		NA		NA		NA
8-Chloro-7-hydroxycoumarin		0.70		0.74		0.83		0.99
3,8-Dichloro-7-hydroxycoumarin		0.67		0.78		0.85		0.98
7-Chloro-6-hydroxy-4-methylcoumarin		0.70		0.73		0.81		0.94

NA: not applicable.

### 2.4 线性范围和方法检出限

分别分析质量浓度为0.01、0.05、0.1、0.2、0.5、1、2.5、5、7.5、10、25、50、100、200 μg/L的香豆素类化合物混合标准溶液,然后根据目标物峰面积进行线性拟合,得到回归方程,具体结果见表3。香豆素类化合物的线性范围为0.05~100 μg/L,线性方程的相关系数(*r*)均大于0.99,线性关系良好。

**表3:** 7种香豆素类化合物的回归方程、相关系数、线性范围以及方法的检出限

Compound	Regression equation	*r*	Linear range/(μg/L)	MDL/(ng/L)
7-Hydroxycoumarin	*y*=1.05×10^6^*x*+9.20×10^5^	0.9996	0.1-100	0.88
6-Hydroxy-4-methylcoumarin	*y*=6.12×10^4^*x*-6.06×10^4^	0.9998	1-100	0.92
6,7-Dihydroxycoumarin	*y*=6.12×10^4^*x*+1.91×10^4^	0.9999	1-100	1.12
Coumarin	*y*=5.16×10^5^*x*+3.01×10^5^	1.000	1-100	0.84
8-Chloro-7-hydroxycoumarin	*y*=1.32×10^5^*x*-9.46×10^4^	0.9996	0.05-100	0.70
3,8-Dichloro-7-hydroxycoumarin	*y*=1.21×10^5^*x*-3.21×10^5^	0.9961	1-100	0.67
7-Chloro-6-hydroxy-4-methylcoumarin	*y*=1.94×10^4^*x*+4.63×10^3^	0.9998	5-100	0.70

MDL: method detection limit; *y*: peak area; *x*: mass concentration, μg/L.

按Qiao等^[18]^介绍的方法向磷酸缓冲液中加入低浓度的标准品,评价7种香豆素的方法检出限(MDL),具体公式如下。


(2) MDL=*t*_(_*_n_*_-__1__,__1__-__∝__=__0__.__99__)_×*S_s_*

式中:*t*_(_*_n_*_-1,1-∝=0.99)_经查表,7次加标试验为3.143; *S*_s_为7次加标试验中测定的标准差。为了能更准确地反应样品中目标物的含量,MDL计算时往往会除以浓缩倍数,经计算7种香豆素的MDL为0.67~1.12 ng/L (见表3)。

### 2.5 加标回收率和精密度

向1 L原水样品中分别加入低(25 ng/L)、中(50 ng/L)、高(100 ng/L)3个水平的7-羟基香豆素、6-羟基-4-甲基香豆素、6,7-二羟基香豆素和香豆素混合标准溶液;1 L出厂水样品中分别加入低(25 ng/L)、中(50 ng/L)、高(100 ng/L)3个水平的8-氯-7-羟基香豆素、3,8-二氯-7-羟基香豆素、7-氯-6-羟基-4-甲基香豆素混合标准溶液。加标后的水样经固相萃取处理,然后考察其加标回收率和相对标准偏差。加标回收率按公式(3)计算。


(3) $$\text{ Recovery }=\frac{A_{3}-A_{0}}{A_{2}-A_{0}}$$

式中:*A*_3_指加标水样固相萃取后香豆素类化合物的峰面积。

结果如表4所示,7种香豆素类化合物的回收率为61.4%~91.5%, RSD为0.9%~11.2%。

**表4:** 7种香豆素的加标回收率和精密度(*n*=6)

Compound	25 ng/L						50 ng/L						100 ng/L			
	Recovery/%		RSD/%				Recovery/%		RSD/%				Recovery/%		RSD/%	
7-Hydroxycoumarin		84.2		6.2				81.7		1.8				88.1		2.0
6-Hydroxy-4-methylcoumarin		83.7		4.2				82.0		5.6				86.6		2.6
6,7-Dihydroxycoumarin		73.7		11.2				71.6		5.2				62.9		3.8
Coumarin		85.0		3.0				89.0		1.7				91.5		2.1
8-Chloro-7-hydroxycoumarin		61.4		1.2				73.6		1.1				74.7		1.2
3,8-Dichloro-7-hydroxycoumarin		73.0		1.4				80.3		0.9				89.3		2.0
7-Chloro-6-hydroxy-4-methylcoumarin		61.9		2.0				76.0		1.2				73.2		1.3

### 2.6 实际样品分析

香豆素可以诱导鱼类的雌激素反应^[7]^,具有一定的生态毒性。一些毒性研究结果发现香豆素类化合物可导致肝脏毒性的产生和肿瘤的发展^[19]^,而且一部分人群更容易受到其毒性的影响^[20]^。这些研究结果表明水体中香豆素类化合物的暴露不仅可威胁生态环境安全,还对健康有一定的危害。相关研究发现水体中的香豆素在紫外线照射下可与氯化物反应,产生毒性更强的氯化衍生物,而且香豆素的矿化程度会明显减少^[21]^,这说明经典的紫外处理不能有效地去除水体中的香豆素类化合物。因此监测饮用水原水中香豆素类化合物的含量,对于评价香豆素在饮用水处理过程中的变化情况及人群暴露情况具有重要意义。本研究中分析了南京市某饮用水处理厂的原水、混凝处理后水样、炭滤处理后水样、砂滤处理后水样以及消毒后的出厂水中7种香豆素类化合物的检出情况(见表5)。实际水样分析发现,7-羟基香豆素、6,7-二羟基香豆素、香豆素的检出率为100%,含量为0.21~27.9 ng/L,可能是因为这几种香豆素在实际水样中含量更为广泛,这在Liu等^[6]^的研究中已经得到证实。氯代香豆素在消毒前的水样中均未被检测,仅8-氯-7-羟基香豆素在出厂水中被检出,含量为0.28 ng/L。该结果也反映出氯代香豆素在饮用水消毒过程中形成。7-羟基香豆素、6,7-二羟基香豆素、香豆素在各种水样中的含量关系为原水>混凝>炭滤,这主要是因为一方面混凝处理能够有效地去除原水中的腐殖质和芳香族前体物^[22,23]^,而后续的炭滤处理可进一步减少它们的含量,因为使用时间相对较短(3年)的活性炭可吸附其中的前体物。这与饮用水处理厂中混凝和炭滤等处理过程可有效减少水体中的取代二苯胺类抗氧化剂一致^[18]^。6-羟基-4-甲基香豆素只在砂滤和出厂水中被检出,砂滤后水样中7-羟基香豆素、6,7-二羟基香豆素、香豆素的含量高于炭滤后水样,这主要是由于砂滤装置使用时间较长(10年),砂滤器中微生物附着量更大,代谢更旺盛,释放的可溶性微生物副产物(SMP)更多^[24⇓-26]^。出厂水中只有6-羟基-4-甲基香豆素的含量高于砂滤后水样,可能的原因为消毒过程中有溶解性有机物转化为6-羟基-4-甲基香豆素。7-羟基香豆素、6,7-二羟基香豆素、香豆素在出厂水中的含量低于砂滤后的水样主要是因为消毒过程中的消耗。8-氯-7-羟基香豆素以外的氯代香豆素没有被检出,可能的原因是这些氯代香豆素前体物的反应活性有差异。以上测量结果与真实水厂中炭滤和砂滤使用年限符合情况较好,反映出本研究建立的分析方法测量结果准确、可靠。该方法可用于监测香豆素类前体物和消毒副产物在水处理过程中的变化情况,并发现水处理过程中存在的问题,可准确地评估人群暴露的风险,为保障饮用水安全提供一些方法支持。

**表5:** 实际水样中香豆素的含量

Compound	Contents/(ng/L)				
	Source water	Coagulation	Carbon filter	Sand filter	Finished water
7-Hydroxy coumarin	1.76	0.92	0.27	0.89	0.21
6-Hydroxy-4-methylcoumarin	-	-	-	1.79	4.69
6,7-Dihydroxy coumarin	1.99	1.87	1.83	27.9	2.81
Coumarin	4.89	3.72	2.83	3.21	2.47
8-Chloro-7-hydroxycoumarin	-	-	-	-	0.28

-: not detected.

## 3 结论

本研究建立了一种固相萃取结合高效液相色谱-三重四极杆质谱法分析实际水样中7种香豆素类化合物的方法,该方法能够准确地分析饮用水处理厂净水单元对水体中香豆素含量变化的影响,可为探究饮用水中香豆素的来源与转化、氯代香豆素形成提供方法支持,可对饮用水处理厂的设备运行效果进行反馈。后期可基于该研究中建立的样品前处理方法,用高分辨质谱对水样进行非靶标分析,有望全面筛查饮用水中香豆素类化合物。
